# Fabrication of Ultranarrow Nanochannels with Ultrasmall Nanocomponents in Glass Substrates

**DOI:** 10.3390/mi12070775

**Published:** 2021-06-30

**Authors:** Hiroki Kamai, Yan Xu

**Affiliations:** 1 Department of Chemical Engineering, Graduate School of Engineering, Osaka Prefecture University, Sakai, Osaka 599-8570, Japan; k.hiroki.71011.3210@gmail.com; 2 Japan Science and Technology Agency (JST), PRESTO, Kawaguchi, Saitama 332-0012, Japan; 3 NanoSquare Research Institute, Research Center for the 21st Century, Organization for Research Promotion, Osaka Prefecture University, Sakai, Osaka 599-8570, Japan

**Keywords:** nanofluidics, narrow nanochannels, nano-in-nano integration, nanogap, zeptoliter

## Abstract

Nanofluidics is supposed to take advantage of a variety of new physical phenomena and unusual effects at nanoscales typically below 100 nm. However, the current chip-based nanofluidic applications are mostly based on the use of nanochannels with linewidths above 100 nm, due to the restricted ability of the efficient fabrication of nanochannels with narrow linewidths in glass substrates. In this study, we established the fabrication of nanofluidic structures in glass substrates with narrow linewidths of several tens of nanometers by optimizing a nanofabrication process composed of electron-beam lithography and plasma dry etching. Using the optimized process, we achieved the efficient fabrication of fine glass nanochannels with sub-40 nm linewidths, uniform lateral features, and smooth morphologies, in an accurate and precise way. Furthermore, the use of the process allowed the integration of similar or dissimilar material-based ultrasmall nanocomponents in the ultranarrow nanochannels, including arrays of pockets with volumes as less as 42 zeptoliters (zL, 10^−21^ L) and well-defined gold nanogaps as narrow as 19 nm. We believe that the established nanofabrication process will be very useful for expanding fundamental research and in further improving the applications of nanofluidic devices.

## 1. Introduction

Nanofluidics involves the study of fluids at nanometer dimensions [1,2,3,4,5,6]. Historically, nanofluidics has been a niche and dormant field. Although the term “nanofluidics” has rarely been used for decades, issues pertaining to nanofluidics have been addressed by researchers under the umbrella of colloid science, membrane science, and chemical engineering. With the advent of chip-based nanofluidic devices (hereafter referred to as “nanofluidic devices”), which belong to the class of planar solid-state transparent devices containing in-plane nanochannel structures, nanofluidics has been garnering significant attention recently in a wide range of disciplines, such as physics, chemistry, biology, medicine, pharmaceuticals, energy, process engineering, material science, and information sciences. This is because nanofluidic devices offer available experimental platforms that enable the research of nanofluidics with various backgrounds, and new devices and approaches are being developed gradually. Further, the use of novel nanofluidic devices has led to the observation of novel physical phenomena and unusual effects caused by fluids confined in nanoscale spaces, including non-linear transport phenomena such as ion current rectification [3,7,8,9,10] and concentration polarization [11,12,13,14], and changes in the liquid properties of water such as lower electric permittivity [15,16], higher proton mobility [17,18,19], and higher viscosity [15,16,20], than those observed in the bulk scales. These phenomena and effects, possibly stemming from ultra-high surface-to-volume ratios and electric double layer overlap featured in confined nanoscale spaces, have recently received widespread attention, but in most cases have remained unexplored. Therefore, while the discipline of nanofluids is not new, it is still in its infancy. Understanding and utilizing these phenomena and effects offers novel mechanisms, powerful tools, and impactful applications that could revolutionize chemistry, biology, material sciences and other related fields. However, several attempts toward such purposes have been severely hampered by the limited choice of available substrate materials, which restricts the fabrication of novel nanofluidic devices.

Nanochannels are the core components of most nanofluidic devices. The methods for fabrication of nanochannels vary depending on the substrate material. While several kinds of substrate materials such as glass, polydimethylsiloxane (PDMS), and plastics are used in the fabrication of microfluidic devices, the widely successful congener of the nanofluidic devices, glass, and particularly fused-silica (amorphous form of silica), is currently the sole substrate material that is ideal for the fabrication of nanofluidic chips. While silicon was initially used for fabrication of nanochannels by the direct use of well-established nanofabrication technologies for microelectronics [21], its optically-opaque nature greatly hindered widespread applications of nanofluidics. The recent years have witnessed the development of methods for the successful fabrication of nanochannels in glass substrates. Driven by such successes and the excellent properties of glass that are favorable for chemical and biological studies and applications, glass has recently become the key substrate material for the fabrication of nanofluidic devices. These excellent properties include superior optical transparency, thermal stability, chemical/biological inertness, mechanical robustness, and hydrophilic nature, all of which are favorable for studies and applications in a wide range of disciplines and interdisciplinary fields.

Several tools have enabled the fabrication of nanochannels in glass substrates. Standard photolithography incorporated with short-time wet or dry etching allows the fabrication of fused silica nanochannels with micrometer-scale widths and nanometer-scale depths, which are, respectively, defined by the practical resolution limits of photolithography and etching duration. Such nanochannels with large widths are also called planar nanochannels or one-dimensional (1D) nanochannels and have been employed in early studies for DNA extension [22]. In contrast, both electron beam lithography (EBL) coupled with dry etching [3,23,24,25,26] and focused ion beam (FIB) milling [27,28,29] are the two major nanofabrication technologies used currently for the fabrication of glass nanochannels in glass substrates. The prominence of these technologies is ascribed to their ability to fabricate the width and depth of glass nanochannels at nanoscales to generate square or two-dimensional (2D) nanochannels. Notably, FIB milling allows the fabrication of narrow square nanochannels with width and depth dimensions as low as tens of nanometers [28,29]. However, FIB milling has several drawbacks, including the low efficiency originating from the direct milling principle [30], charging effect caused by ion irradiation on the insulating substrate, coarse channel wall surfaces resulting from the simultaneous deposition effect, and the difficulty in maintaining stable processing conditions, particularly during milling over a long duration and/or a large area. Unfortunately, these drawbacks render the controllable, reproducible, and predictable high-throughput fabrication of glass nanochannels using FIB milling highly challenging, thus hindering the wider application of the technology for the fabrication of nanofluidic devices. In contrast, EBL coupled with dry etching exhibits overwhelming advantages over FIB milling, owing to its well-defined principle based on fine electronic, chemical, and physical mechanisms. Such advantages are beneficial in the efficient fabrication of square nanochannel structures with high controllability, reproducibility, and predictability, as reported in several studies [3,31,32]. Despite its theoretical potential to create nanopatterns with linewidths as narrow as tens of nanometers [33], in practice, achieving this on glass by using EBL coupled with dry etching remains highly challenging due to its non-crystalline property (which is unfavorable for top-down fabrication of ultasmall nanostructures) and the need to control several complicated processes. As a result, most current studies on nanofluidic devices are based on the use of glass nanochannels with linewidths above 100 nm. Nanofluidics can potentially exploit the aforementioned new phenomena and unusual effects at nanoscales, and in most cases, below 100 nm [2,5,6]. Therefore, the fabrication of nanochannel structures with narrow linewidths of several tens of nanometers in glass substrates using EBL coupled with dry etching is critical for the advancement of the field of nanofluidics.

In this study, we achieved efficient, accurate, and precise-fabrication of fine ultranarrow nanochannels with feature sizes of several tens of nanometers by focusing on two key processing parameters, the EB resist thickness and development time, among the various complicated nanofabrication processing parameters of the EBL coupled with dry etching processes. The use of an optimized nanofabrication process makes it possible to fabricate fine ultranarrow nanochannels and nanochannels with similar or dissimilar material-based ultrasmall nanocomponents. The fabrication process established in this study will aid the advancement of fundamental research and the further improvement of nanofluidic device-based applications.

## 2. Experimental

### 2.1. Basic Processes of Nanofabrication

Nanofabrication was performed by combining nanopattern transfer with subtractive techniques (such as EBL and plasma dry etching) and/or processes of nanopattern transfer with additive techniques (such as physical vapor deposition and lift-off). These individual processes are briefly described herein, and further details are available in the literature [15,34,35,36].

#### 2.1.1. EBL

An EBL process was used for transferring nanopatterns, generated via computer-aided-design (CAD), onto a substrate. First, an EB resist (ZEP520A; ZEON, Tokyo, Japan) was spin-coated on a perfectly cleaned fused-silica glass substrate (30 mm × 40 mm × 0.7 mm; Sendai Quartz, Sendai, Japan) using a spin coater (MA-A100; MIKASA, Tokyo, Japan). To investigate the effect of the thickness of the EB resist on nanofabrication, different spin-coating speeds of 2500, 3000, 4000, 4300, and 4500 rpm were applied to obtain EB resist layers with different thicknesses, which were further measured using a stylus surface profiler (Dektak 150; Bruker, MA, USA). Next, an electron beam under a standard beam condition was irradiated onto the EB resist-coated glass substrate using an electron beam lithography system (ELS-7500EX; ELIONIX, Tokyo, Japan). Finally, the EB irradiated-substrate was developed in xylene (Wako Special Grade; Wako, Osaka, Japan) in a thermostatic bath (TR-2AR; AS ONE, Osaka, Japan) at 25.0 ± 0.1 °C, and the resulting designed nanopattern was transferred to the EB resist layer.

#### 2.1.2. Plasma Dry Etching

To transfer the EB resist nanopattern onto the glass substrate, a plasma dry etching process was applied using a reactive ion etching (RIE) system (RIE-10NR; SAMCO, Kyoto, Japan). In the etching process, fluorine gas (Kanto Denka Kogyo, Tokyo, Japan) was used as the working gas to achieve an etching rate of 22–26 nm/min. After etching, the remaining resist was removed using a mixture (3/1, v/v) of dimethyl sulfoxide (99.0%; Wako, Osaka, Japan) and xylene.

#### 2.1.3. Physical Vapor Deposition and Lift-Off

Physical vapor deposition processes and lift-off were used to fabricate the gold nanopatterns. After transferring the computer-aided nanopattern designs to the resist layer on the substrate using the standard EBL process, 5 nm-thick chromium (Cr, 99.9%; Nilaco, Tokyo, Japan) and 30 nm-thick Au (99.99%; Tanaka Kikinzoku Kogyo, Tokyo, Japan) films were sequentially deposited on the substrate using vacuum evaporation equipment (A9858; Seinan Industries, Osaka, Japan) at 10^−5^ Pa. Here, the thin Cr layer played a role in increasing the adhesion between the gold layer and the glass substrate. After the process for removing the Au/Cr on the resist using a mixture of dimethyl sulfoxide and xylene (3/1, v/v), the remaining Au/Cr parts formed gold nanopatterns on the substrate (i.e., lift-off).

### 2.2. Fabrication and Characterization of Nanochannel Structures

Two types of 50 parallel 2D nanochannel structures (40 nm wide, 30 nm deep, and 300 μm long, as targeting values) with and without pockets (30 nm × 30 nm and 60 nm × 30 nm, 30 nm deep, as targeting values) were fabricated using the above-mentioned processes of EBL and plasma dry etching. This is illustrated in Figure S1 in Electronic Supplementary Information (ESI). The obtained nanochannel structures were characterized using a field-emission scanning electron microscope (FE-SEM; SU800, Hitachi High-Tech, Tokyo, Japan), and the channel widths of 13 locations in each of the 20 nanochannels were measured at equal intervals. The depth of the nanochannel structures was measured using a stylus surface profiler, and the surface roughness was measured using an atomic force microscope (AFM; AFM5200S, Hitachi High-Tech, Tokyo, Japan).

### 2.3. Fabrication and Characterization of Gold Nanogaps

Gold nanogap structures (100 × 100 nm squares, spaced by gaps of 30 nm, as targeting values) were fabricated using the above-mentioned processes of EBL, physical vapor deposition, and lift-off, and this is illustrated in Figure S2 in ESI. The obtained gold nanogaps were characterized by FE-SEM.

### 2.4. Fabrication and Characterization of Nano-In-Nano Structures 

The nano-in-nano structures were fabricated using a multiple-step EBL process (Figure S3 in ESI) sustained by a high-precision placement control technique previously developed by us [15,34]. A brief description of the fabrication process is as follows. First, a pair of cross-shaped marks comprising gold/chromium (Au/Cr, 30 nm/5 nm thick) was fabricated on a glass substrate using EBL (i.e., 1st EBL), physical vapor deposition, and lift-off. The cross-shaped marks were several tens of nanometers wide and several hundreds of nanometers long, and were used as reference marks for detecting the location of the glass substrate during the following two EBL steps. This is because the placements of the cross-shaped marks can be precisely detected by scanning with an electron beam (EB). Then, nanochannels were fabricated on the glass by a second EBL with accurate placement using the reference marks, dry etching, and EB resist removal. Finally, using the reference marks again, gold nanogaps (Au/Cr, 30 nm/5 nm thick) were fabricated in the nanochannels using a third EBL, deposition, and lift-off. The fabricated nano-in-nano structures were characterized using FE-SEM.

### 2.5. Fabrication and Characterization of Nanofluidic Devices

Microchannels were dry-etched on a fused-silica glass substrate after photolithography and the resulting inlet and outlet holes were penetrated using a diamond-coated drill [36]. The nanofluidic chip was obtained by bonding the two substrates comprising micro- and nanochannels with ultrasmall gold nanogaps (i.e., nano-in-nano components) according to a gold-pattern-friendly bonding process previously reported by us [15,23,34]. 

## 3. Results and Discussion

### 3.1. Parameter Investigation for Optimization of Chip-Based Nanofabrication Process

**Figure 1:**
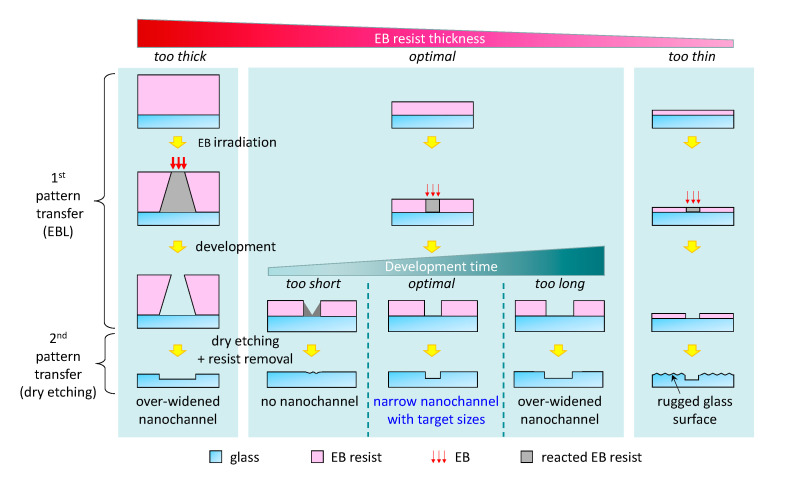
Schematic of the difference in structures of nanochannels fabricated under different conditions of varying EB resist thickness and development time.

EBL and the ensuing plasma dry etching processes involve several intricate parameters, such as the resist layer thickness, dose time, voltage and beam current of the electron beam, field size and number of dots, development time, plasma density, etching rate, etching selectivity, etching time, etc. All of these parameters are involved in the nanofabrication process; however, in this study, we focused on the resist layer thickness and development time, considering the required multiple processes in chip-based nanofabrication, which is different from the general processes for each individual nanopattern transfer steps, as described below (Figure 1).

The chip-based nanofabrication process involves two transfer steps (Figure 1). The first is EBL transfer, which refers to the transfer of the CAD data onto the EB resist surface, and the second is the plasma dry etching transfer, which involves moving the EB resist pattern onto the glass surface. In general, for the fabrication of EB resist patterns with features of the size of tens of nanometers, extremely thin EB resist layer thicknesses are needed because when the EB resist layer thickness is high, the irradiating electron beam (EB) scatters on the EB resist layer and the resist pattern-spreads during the first EBL transfer (Figure 1). However, chip-based nanostructures must be considered for the negative effect of the second transfer. In the second transfer, the EB resist uses as a glass protection from plasma dry etching and is etched along with the glass. Therefore, when the resist layer is too thin, there is a risk that the non-nanochannel glass area is also etched which results in the ruggedness of the surface (Figure 1). Rugged glass surfaces are not favorable because they cause failures in chip bonding, which is the final step of nanofluidic device fabrication [32,36]. Therefore, the EB resist layer should not be too thin. For these reasons, investigation for determining the appropriate EB resist thickness that is neither thin nor thick is needed for fabricating chip-based structures with feature sizes of tens of nanometers (Figure 1).

To determine the optimal resist thickness, the selection ratio of the resist to glass during plasma dry etching (second transfer) needs to be examined. In this study, the EB resist solution was diluted three-fold to achieve a sufficiently thin EB resist layer thickness. As revealed by preliminary experiments, the resist-to-glass selection ratio of etching (defined as the ratio between the etching rate of resist and the etching rate of glass) was approximately 1/1 for the case using three-fold diluted resist. Considering that our target depth of nanochannels was 35–40 nm and the thickness of the resist layer may not be uniform in the range of tens of nanometers, we determined the target EB resist thickness to be 70–80 nm to sufficiently prevent the glass area without nanochannels from being etched, according to our experience.

**Figure 2:**
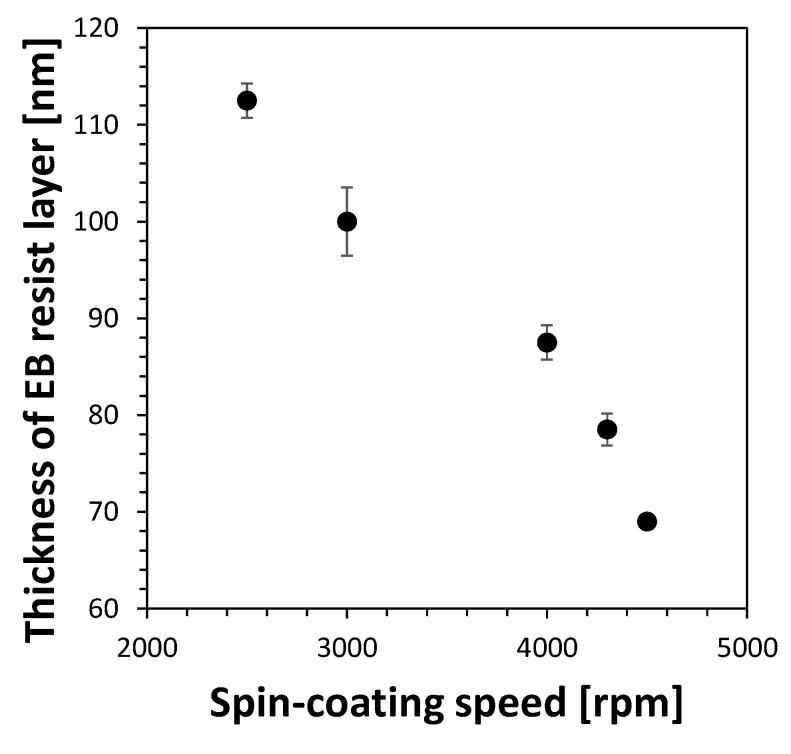
Relationship of the thickness of EB resist layer vs. spin-coating speed.

Figure 2 shows the thicknesses of the EB resist layers fabricated using a spin coater with varying spin-coating speeds (2500, 3000, 4000, 4300, or 4500 rpm). The EB resist thicknesses indicated different heights between the glass surface areas coated and uncoated with the EB resist when measured using the stylus surface profiler. As a result, a negative linearity was observed between the spin-coating speed and EB resist layer thickness, indicating that a thinner EB resist layer thickness could be obtained by increasing the spin-coating speed (Figure 2). The minimum EB resist layer thickness was 69 nm (rotation speed of 4500 rpm), but as mentioned above, this condition presents a high risk for the etching of the glass area without nanochannels (Figure 1). On the other hand, a thickness of 78 nm (rotation speed of 4300 rpm) presented a reduced risk and matched the target value (70–80 nm). Therefore, the optimal rotation speed was determined to be 4300 rpm, which delivered a 78 nm EB resist layer thickness. 

However, to fabricate chip-based structures with feature sizes of tens of nanometers, investigation of the EB resist thickness is not sufficient. The development time, which is the time required to remove the EB-irradiated parts of the resist by dissolving in xylene, must also be investigated. The development time significantly affects the size of the EB resist patterns that form the nanochannels in the next transfer, and the optimal time depending on the EB resist thickness should be considered (Figure 1). Significantly shorter development times prevent the removal of the EB-irradiated resist from the substrate (Figure 1). On the other hand, longer development times broaden the developing range than that of the desired pattern (Figure 1). This is because of the widening of the irradiated portion due to electron scattering, even if the resist layer thickness is thin. Therefore, adequate development time and the appropriate EB resist layer thickness are critical parameters for the successful fabrication of nanostructures with sizes in the order of tens of nanometers. 

**Figure 3:**
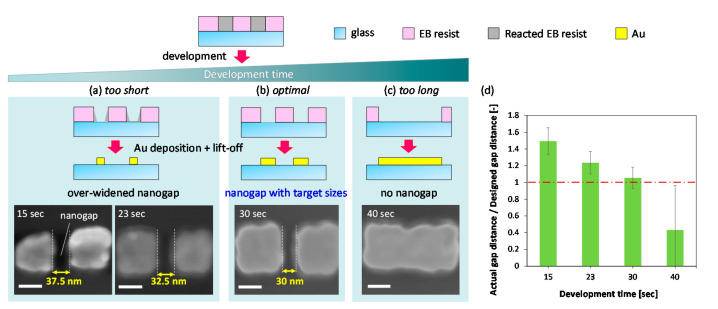
Schematic of influence of developing time on gap distance in the fabrication of gold nanogaps and representative SEM images of gold nanogaps fabricated at different development times, corresponding to cases of (**a**) too short (15 s and 23 s), (**b**) optimal (30 s), and (**c**) too long (40 s) development times, respectively. White scale bar is 100 nm. (**d**) Relationship between actual gap distance/designed gap distance of gold nanogaps and development time.

To investigate the influence of development time, the fabrication of small gold nanogaps at different conditions of different development times were studied (Figure 3 and Figure S2). The gold nanogaps were used instead of glass nanochannels because of the features of the nanostructures can be fabricated easily, efficiently, and accurately, and can be observed and characterized by FE-SEM, thereby being favorable for numerous experimental studies required in parameter investigation. FE-SEM has been extensively used to observe and characterize small nanostructures; however, in principle, the samples (at least at the surface) must be electrically conductive. When scanned by an electron beam, nonconductive samples such as glass substrates used in this study accumulate electrostatic charge, which interferes with the scanning and causes various image artifacts. Hence, it is hard to directly observe the ultrasmall nanostructures in glass substrates by using FE-SEM. Coating of electrically-conductive materials by deposition or sputtering is an effective method to improve the electrical conductivity of surfaces of non-conducting materials for FE-SEM imaging. The method, however, is not favorable for this study. Although the additional conductive layer is thin, its thickness is sufficient for hiding the details of the ultranarrow nanochannels with linewidths in order of tens of nanometers, making it difficult to accurately characterize them. In contrast, the gold nanogaps can be used as ideal samples in parameter investigation for efficient and arcuate FE-SEM observation and characterization, owing to their excellent electrical conductivity. While the fabrication of gold nanogaps employs different mechanisms and processes for the second pattern transfer (additive transfer based on physical vapor deposition) compared to those for nanochannels, the ability to define an EB resist nanopattern does not change; therefore, the optimized parameter conditions obtained by using the gold nanogap investigation can be applied to the fabrication of nanochannels in the glass substrates. In this study, we used the distance of the gold nanogap as a parameter to investigate the influence of development time. 

The results of the optimization of the development time on the gold nanogap fabrication are shown in Figure 3. The representative gold nanogaps (design value: 100 nm × 100 nm squares, gap distance 30 nm) fabricated at each development time (15, 23, 30, and 40 s) were observed and characterized using FE-SEM (Figure 3a–c). The results revealed that the gap distance became smaller with longer development time and disappeared beyond a certain time point. At 40 s, which is beyond the optimal development time, several places were observed where the gold gap were not well formed (Figure 3c). In Figure 3d, the actual gap distance/design gap distance represents the ratio between the actual gold nanogap distance and the designed gold nanogap distance (30 nm). The gold nanogaps with target sizes are considered fabricated in the case where the ratio is close to 1.0. At 30 s, the ratio was closest to 1.0, which indicates that 30 s was the optimal development time (Figure 3b,d). On the other hand, at 40 s, the ratio was well below 1.0 and its standard deviation (SD) was quite large (Figure 3d), due to the fact that gold nanogaps were not well formed at many places in this case as shown in Figure 3c. These results revealed that a development time of 30 s was optimal for fabricating gold nanogaps and nanochannels with feature sizes of several tens of nanometers on the glass substrate.

### 3.2. Fabrication and Characterization of Ultranarrow Nanochannels

**Figure 4:**
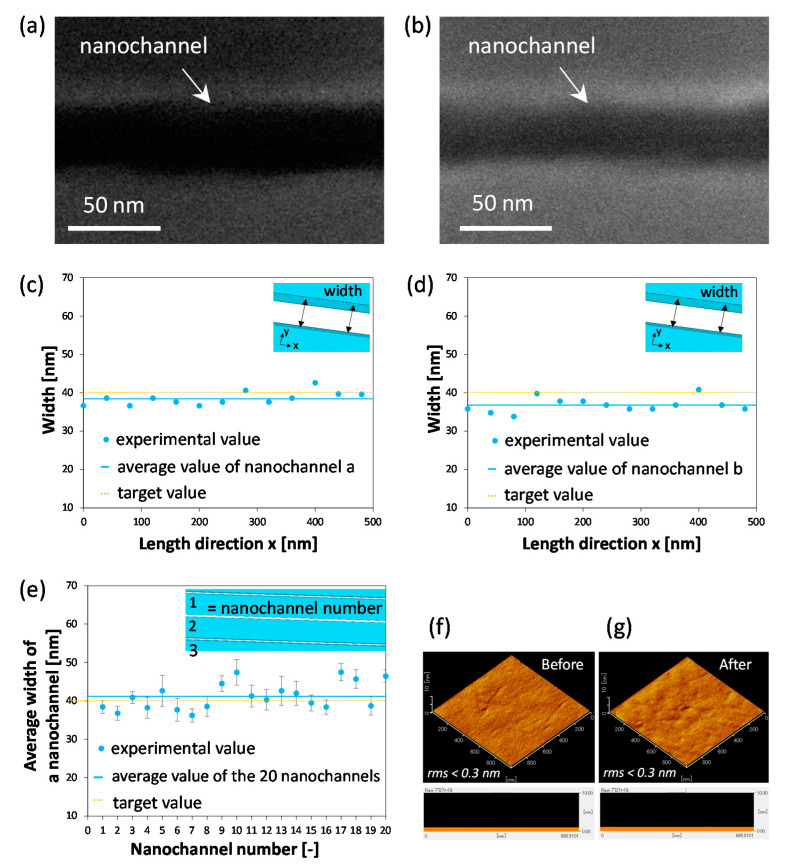
(**a**,**b**) SEM images of representative ultranarrow nanochannels (32 nm deep) fabricated in glass substrates. (**c**,**d**) Variation of the width along the length direction of ultranarrow nanochannel shown in (a, b), respectively. (**e**) Variation of the average widths between 20 ultranarrow nanochannels (*n* = 13 for each nanochannel). AFM images of glass substrate (**f**) before and (**g**) after nanofabrication.

The fabrication of ultranarrow nanochannels with tens of nanometer linewidths were achieved by using the optimized conditions, with a resist layer thickness of 78 nm and a development time of 30 s. As a demonstration, the fabrication of ultranarrow nanochannels with a channel width of 40 nm as a target value was performed. Figure 4a,b show representative SEM images randomly extracted from the fabricated ultranarrow nanochannels (depth of 32 nm, measured by a stylus surface profiler). It should be noted that the substrate after nanofabrication was spin-coated with a conductive polymer aqueous solution of poly (isothianaphthenediyl sulfonate) and additives, i.e., ESPACER^®^ (Showa Denko, Tokyo, Japan), to improve electrical conductivity to some extent for FE-SEM imaging. 

To characterize the lateral line uniformity of the fabricated nanochannel, the width of each representative nanochannel (Figure 4a,b) was measured for every 40 nm at 13 locations (Figure 4c,d). In Figure 4c,d, the horizontal axis shows a distance x [nm] from the left end of the measurement and the vertical axis shows the nanochannel widths at that distance x [nm]. Also, the yellow dotted line represents the target value of 40 nm, and the blue line represents the average widths of the fabricated nanochannels shown in Figure 4a,b measured at 13 locations in the 0–480 nm range (hereafter called “the average widths of nanochannel a” and “the average widths of nanochannel b”). The results showed that the average widths of nanochannel a and b were 38.4 ± 1.7 nm and 36.8 nm ± 1.8 nm, respectively, which were very close to the 40 nm target value. In addition, for both cases the SD values of the width are less than 2.0 nm, indicating that the fabricated ultranarrow nanochannels had quite high uniformity of the lateral line. Furthermore, the widths of the 20 representative nanochannels were measured at 13 points every 40 nm for each. In Figure 4e, the horizontal axis shows the nanochannel number of the measured 20 nanochannels (each of 20 nanochannels is numbered 1 to 20 in series, and are referred as nanochannel 1, 2, 3, and so on) and the vertical axis shows the average widths of each numbered nanochannel. Also, the yellow dotted line represents the target value of 40 nm, and the blue line represents the average widths of the 20 nanochannels. As a result, the average widths of the 20 nanochannels was 41.2 nm ± 3.5 nm, which was also very close to the target value and thus reveals that the accuracy of the fabrication was quite high. In addition, the small standard deviation (3.5 nm) suggests that there is no significant difference in the average width among those nanochannels, implying that the precision of the fabrication was also high. 

Due to the ultrahigh surface-to-volume ratios of nanochannels, surface morphology of the nanochannels dominate a variety of nanofluidic phenomena. Smooth nanochannel wall is usually favorable and desired for a variety of applications of nanofluidic devices. Hence, characterization of surface morphology of the fabricated nanochannels is important. Due to the ultra-narrowness of the fabricated nanochannels in this study, it is difficult to directly characterize the wall surface of the nanochannels by using AFM, which is a powerful tool to characterize surface morphology. This is because the micrometer sized cantilever of AFM is significantly larger than the width of the fabricated narrow nanochannels, making it difficult to measure the inner walls of the nanochannels. Thus, in this study, together with the narrow nanochannels, a wide microchannel (145 μm wide, 32 nm deep) was simultaneously fabricated in the same glass substrate under the same conditions and was used for indirectly obtaining the morphological information of the fabricated nanochannels by using AFM. The results (Figure 4f,g) revealed that both the morphologies of glass surfaces before and after the nanofabrication were homogeneous and exhibited no significant difference, suggesting that the fabrication process did not cause adverse effects in the morphology of the glass surface. The root-mean-square roughness (RMS) values before and after etching were less than 0.3 nm (Figure 4f,g), indicating both surfaces were very smooth. 

Therefore, the optimized fabrication process allows the fabrication of fine nanochannels with ultranarrow linewidths of several tens of nanometers, uniform lateral features, and smooth morphologies, in an accurate and precise way. In addition, considering the significantly shorter processing time of EBL coupled with dry etching used in this study (e.g., EB irritation time was 7.6 min and etching time was 1.3 min for 50 ultranarrow nanochannels) than that of the FIB milling process (requires several hours even when fabricating one nanochannel [30]), the optimized fabrication process would be an efficient way for the ultranarrow nanochannel fabrication. 

### 3.3. Fabrication and Characterization of Ultranarrow Nanochannels with Ultrasmall Nanocomponents

**Figure 5:**
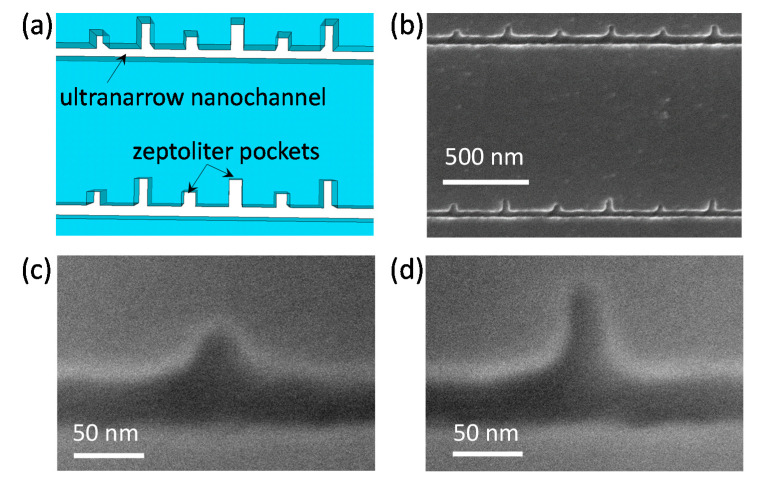
(**a**) Schematic and (**b**) SEM images of ultranarrow nanochannels with zeptoliter (zL, 10^−21^ L) pockets. SEM images of nanochannels with ultrasmall (**c**) square pocket and (**d**) rectangular pocket at a large magnification of 200,000×.

While standard straight nanochannels are widely used in fundamental studies of nanofluidics, nanochannels with nanocomponents are strongly desired especially in the development of potential nanofluidic applications. The optimized fabrication process also allows the fabrication of ultranarrow nanochannels with ultrasmall nanocomponents. As a demonstration, we fabricated arrayed ultranarrow nanochannels (40 nm-wide) with square (30 nm × 30 nm, as targeting values) and rectangular (60 nm × 30 nm, as targeting values) pockets with zeptoliter volumes (zL, 10^−21^ L) (Figure 5a). 

The substrate after nanofabrication was also spin-coated with ESPACER^®^ to improve electrical conductivity for FE-SEM imaging (Figure 5b). The details of the two types of pockets were further characterized using FE-SEM at a large magnification of 200,000×, as shown in Figure 5c,d. While both types of pockets exhibited broadened openings with the side connected to the nanochannel being 71.1 nm wide for the square pocket and 78.6 nm wide for the rectangular pocket, and narrowed ends with the side opposite to the nanochannel being 19.8 nm wide for the square pocket and 18.5 nm wide for the rectangular pockets in comparison with those of the targeted value (30 nm), the lengths which indicate the distance between the opening and the end were 34.5 nm for the square pocket and 66.3 nm for the rectangular pocket. The full width at half maximum (FWHM), which is the width at the half length between the opening and the end on the *Y*-axis, was 37.8 nm for the square pocket and 34.1 nm for the rectangular pocket, and the values for both types of pockets were quite close to the targeted value. In addition, the volumes were 42 zL and 77 zL for the square pocket and the rectangular pocket, respectively, according to a calculation based on the actual contours and the depth (32 nm) of both types of pockets. Considering that the current studies using nanofluidic structures operate mainly with volumes at the femtoliter (fL, 10^−15^ L) to attoliter (aL, 10^−18^ L) levels, the use of such ultrasmall pockets hold potential for further extension of nanofluidics to the zL regimes in the future.

### 3.4. Fabrication and Characterization of Nanochannels Integrated with Ultrasmall Gold Nanogaps

**Figure 6:**
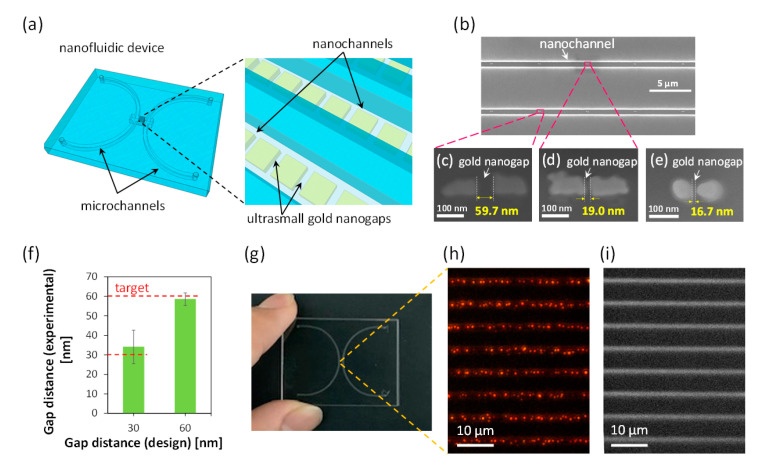
(**a**) Schematic of a nanofluidic device with ultrasmall gold nanogaps in nanochannels. (**b**)SEM image of nanochannels with ultrasmall gold nanogaps. (**c**–**e**) SEM images of representative gold nanogaps. (**f**) Relationship between target value and average of the experimental value of the gold nanogap distance (n = 11 for each). (**g**) Photo of fabricated nanofluidic device. (**h**) Bright-field microscopic image of bonded nanochannels with ultrasmall gold nanogaps. (**i**) Fluorescent microscopic image of nanochannels with ultrasmall gold nanogaps after the introduction of rhodamine B solution (9.8 μM).

The integration of functional (e.g., chemical, biological, optical, electrical, magnetic, thermal, etc.) components of dissimilar materials in nanochannels would open new avenues in the fusion of nanofluidics with a variety of other fields. The fabrication of nanochannels integrated with ultrasmall dissimilar material-based nanocomponents was further demonstrated by incorporating the optimized fabrication process with the nano-in-nano integration technology previously developed by us [15,34]. The use of nano-in-nano integration technology enables the fabrication of arbitrary patterns of dissimilar materials in a closed, small nanochannel. As a demonstration, we fabricated a nanofluidic device with narrow gold nanogap arrays (20 pairs per nanochannel) in 30 parallel nanochannels (Figure 6a) by taking advantage of the optimized nanofabrication process. Owing to its excellent chemical and physical properties, gold is a universal material which has been employed for fabricating chemical, biological, optical, electrical, and thermal components in a variety of devices at different scales. In addition, gold nanogaps have exhibited a wide range of applications in chemistry, physics, nanotechnology, biology, biotechnology, diagnostics, medicine, photonics, electronics, energy, materials science, and information science. Therefore, we chose the fabrication of gold nanogap arrays in nanochannels as the demonstration.

The fabrication of nanochannels integrated with gold nanogaps of desired distance was achieved (Figure 6b–e). Among them, the minimum distance between the gold nanogaps with well-defined straight gap structures was 19.0 nm, as shown in Figure 6d. We also observed some nanogaps with much smaller gap distances than 19.0 nm (e.g., Figure 6e). However, such nanogaps were mostly formed by round-shaped gold nanopatterns as shown in Figure 6e, probably resulting from local imperfect lift-off of gold which usually takes place in the fabrication of extremely small nanopatterns. In this study, the targets for the gold nanogap distance were 60 and 30 nm. Figure 6f shows the relationship between the target value and the average of the experimental value of the gold nanogap distance. The distances of these nanogaps (n = 11) were measured using FE-SEM. The results show that the average distances of the fabricated gold nanogaps agreed well with the target values, revealing that the optimized nanofabrication processes are also applicable to the fabrication of ultrasmall gold nanogaps in nanochannels. 

The substrate comprising the nanochannels integrated with ultrasmall nanogap arrays was further bonded with another glass substrate comprising two microchannels to form the nanofluidic device (Figure 6g,h). In addition, introduction of the liquid into the nanochannels with ultrasmall gold nanogaps was demonstrated by filling a solution of a fluorescent dye (rhodamine B, 9.8 μM) through an inlet of the nanofluidic device. The liquid in the nanochannels were observed using a fluorescence microscope (BX53, Olympus, Tokyo, Japan) with an electron multiplying charge-coupled device (EM-CCD) camera (iXon Ultra 888, Andor, Oxford Instruments, Belfast, UK). Strong fluorescence ascribed to rhodamine B was detected in the arrayed nanochannels, indicating that the liquid was successfully introduced into the nanochannels having ultrasmall gold nanogaps (Figure 6i). This result suggests that the use of the process established in this study allows the fabrication of nanofluidic devices with ultrasmall components.

## 4. Conclusions

In this study, we established a process for the fabrication of ultranarrow nanochannels and nanochannels with ultrasmall nanocomponents in glass substrates by optimizing the nanofabrication process of the EBL coupled with dry etching. The thickness of the EB resist layer and development time were investigated in detail. The use of the optimized process allowed the efficient fabrication of fine glass nanochannels with sub-40-nm linewidths, uniform lateral features, and smooth morphologies in an accurate and precise way. The established process also enabled the integration of similar or dissimilar material-based ultrasmall nanocomponents in the ultranarrow nanochannels. The fabrication of such nanochannel structures is highly desirable for fundamental- and application-oriented studies in nanofluidics; however, this has remained a challenge. Therefore, we believe that the established fabrication process would prove exceedingly useful for expanding fundamental research and initiate further remarkable applications of nanofluidic devices.

## Supplementary Materials

The following are available online at https://www.mdpi.com/article/10.3390/mi12070775/s1, Figure S1: Schematic of process for fabricating the nanochannel in the glass substrate, Figure S2: Schematic of process for fabricating gold nanogaps on the glass substrate, Figure S3: Schematic of process for nano-in-nano integration guided by the high-precision placement control technique.
