# Supplementary material for: Fabrication of Ultranarrow Nanochannels with Ultrasmall Nanocomponents in Glass Substrates
Source: Micromachines (Basel). 2021 Jun 30;12(7):775. doi: 10.3390/mi12070775 (PMC8305551; doi:10.3390/mi12070775)
Supplement: Supplementary file 1 [file micromachines-12-00775-s001.zip › micromachines-1283504-supplementary.pdf]

micromachines

Article

Fabrication of ultranarrow nanochannels with ultrasmall nano-components in glass substrates

Hiroki Kamai [1] and Yan Xu*,[1,2,3]

[1] Department of Chemical Engineering, Graduate School of Engineering, Osaka Prefecture University, Sakai, Osaka 599-8570, Japan. Email: xu@chemeng.osakafu-u.ac.jp
[2] Japan Science and Technology Agency (JST), PRESTO, Kawaguchi, Saitama 332-0012, Japan
[3] NanoSquare Research Institute, Research Center for the 21st Century, Organization for Research Promotion, Osaka Prefecture University, Sakai, Osaka 599-8570, Japan

* Correspondence: xu@chemeng.osakafu-u.ac.jp; Tel.:

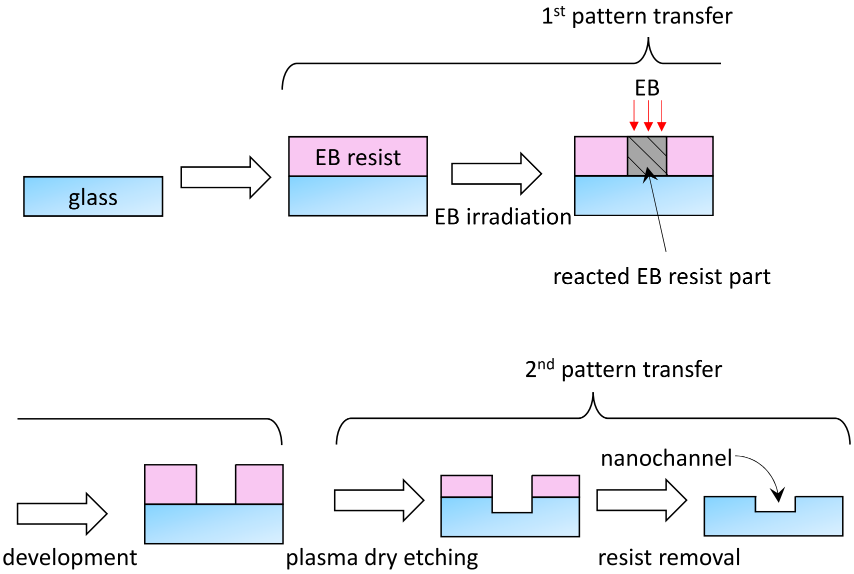

Figure S1. Schematic of process for fabricating the nanochannel in the glass substrate.

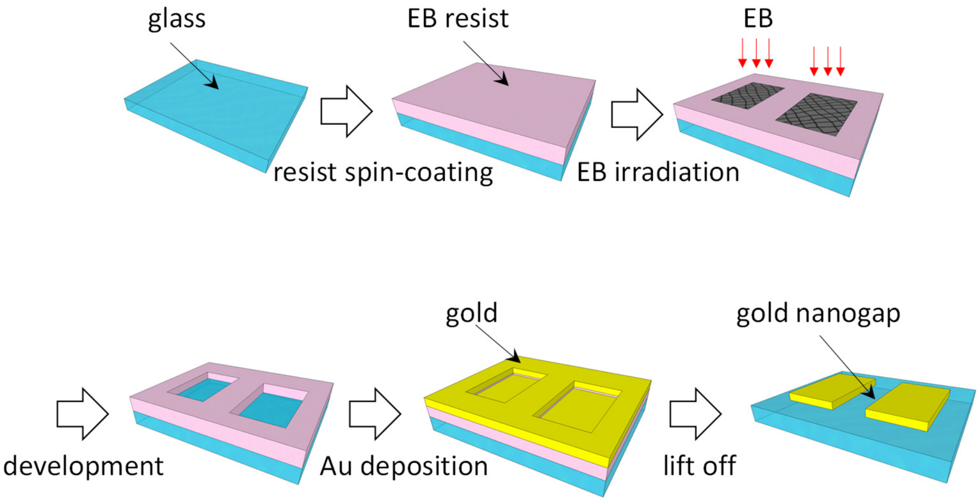

Figure S2. Schematic of process for fabricating gold nanogaps on the glass substrate.

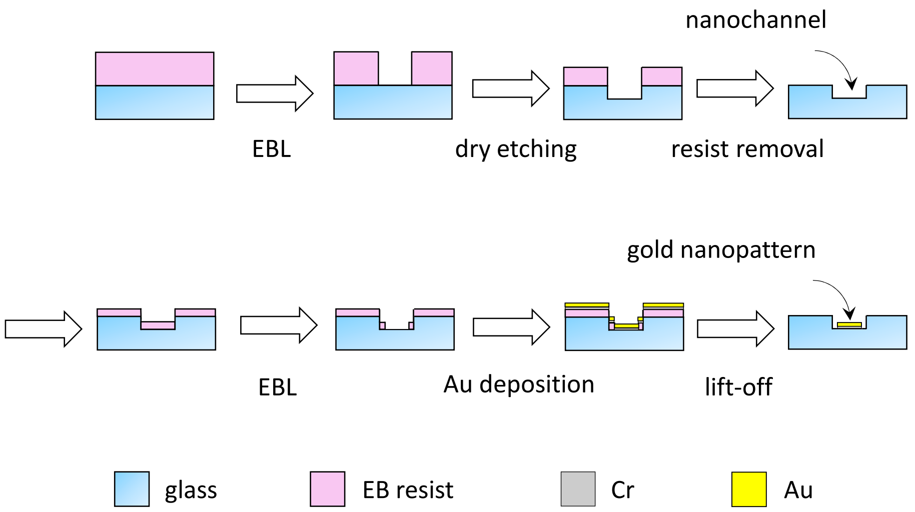

Figure S3. Schematic of process for nano-in-nano integration guided by the high-precision placement control technique.
